# SOX4 inhibits GBM cell growth and induces G0/G1 cell cycle arrest through Akt-p53 axis

**DOI:** 10.1186/s12883-014-0207-y

**Published:** 2014-11-01

**Authors:** Jing Zhang, Huawei Jiang, Jiaofang Shao, Ruifang Mao, Jie Liu, Yingying Ma, Xuefeng Fang, Na Zhao, Shu Zheng, Biaoyang Lin

**Affiliations:** Cancer Institute (Key Laboratory of Cancer Prevention and Intervention, China National Ministry of Education), Second Affiliated Hospital, College of Medicine, Zhejiang University, Hangzhou, Zhejiang Province P R China; Systems Biology Division and Propriumbio Research Center, Zhejiang-California International Nanosystems Institute (ZCNI), Zhejiang University, Hangzhou, Zhejiang Province P R China; Department of Bioinformatics, School of Basic Medical Sciences, Nanjing Medical University, Nanjing, Jiangsu Province P R China; Department of Urology, University of Washington, Seattle, WA 98195 USA

## Abstract

**Background:**

SOX4 is a transcription factor required for tissue development and differentiation in vertebrates. Overexpression of SOX4 has been reported in many cancers including glioblastoma multiforme (GBM), however, the underlying mechanism of actions has not been studied. In this study, we investigated the role of SOX4 in GBM.

**Methods:**

Kaplan-Meier analysis was performed to assess the association between SOX4 expression levels and survival times in primary GBM samples. *Cre/lox P* system was used to generate gain or loss of SOX4 in GBM cells, and microarray analysis uncovered the regulation network of SOX4 in GBM cells.

**Results:**

High SOX4 expression was significantly associated with good prognosis of primary GBMs. SOX4 inhibited the growth of GBM cell line LN229, A172G and U87MG, partly via the activation of p53-p21 signaling and down-regulation of phosphorylated AKT1. Gene expression profiling and subsequent gene ontology analysis showed that SOX4 influenced several key pathways including the Wnt/ beta-catenin and TGF-beta signaling pathways.

**Conclusions:**

Our study found that SOX4 acts as a tumor suppressor in GBM cells by induce cell cycle arrest and inhibiting cell growth.

**Electronic supplementary material:**

The online version of this article (doi:10.1186/s12883-014-0207-y) contains supplementary material, which is available to authorized users.

## Background

The sex-determining region Y (SRY) box (SOX) gene family, characterized by the highly conserved HMG-domain responsible for sequence specific DNA binding, encodes transcription factors that are essential for embryonic development, cell fate determination, differentiation, and proliferation [1]. So far, twenty pairs of SOX genes have been identified in the human and mouse genomes [2].

SOX4 has been found to be over-expressed in adenoid cystic carcinoma (ACC), hepatocellular carcinoma, bladder tumors, acute myeloblastic leukemia, prostate cancer, endometrial cancer and glioblastoma [3-8]. SOX4 was further identified as a common transcription factors for neoplastic transformation and progression in a large-scale meta-analysis of cancer microarray data [9]. However, SOX4’s mode of action in cancer is complicated as SOX4 can act either as an oncogene [4,10,11] or a tumor suppressor [4,12]. As an oncogene, SOX4 overexpression predicts poor outcome of colorectal cancer [13]. Its overexpression in prostate cancer correlated strongly with Gleason score [6]. Knock down of SOX4 induced apoptosis in prostate cancer cells [6] and adenoid cystic carcinoma ACC3 cells [3]. SOX4’s role in bladder is perplexing: SOX4 is over expressed in bladder cancer tissues compared to normal tissues, but strong SOX4 expression was found to be correlated with increased patient survival (P <0.05) of bladder cancer [4], and when introduced to bladder cancer cell line HU609, it reduced cell viability by promoting apoptosis and necrosis [4]. As a tumor suppressor, introduction of SOX4 into hepatocarcinoma Hep3B and HepG2 cells induced apoptosis via the caspase cascade with caspase-1 activation [14]. In HeLa cells, SOX4 was shown to also induce apoptosis via the caspase dependent pathway [15].

Glioblastoma multiforme is the most common and aggressive type of malignant gliomas (WHO grade IV) with an annual incidence of 2 to 3 per 100,000 population [16]. Currently, the standard therapy for gliomas consists of maximal surgical resection, followed by chemotherapy [16]. However, because of its malignant features manifested by fast growth and chemo- or radio-resistance, most of patients die from the recurrence with malignant gliomas within one year [17]. Others and we have showed that SOX4 is a target of TGF-beta signaling and is involved in maintaining stemness of glioma-initiating cells [4,8,18,19]. To further understand the molecular mechanism of SOX4 in GBM, in this study, we systematically studied the function of SOX4 in GBM cells using the *Cre/lox P* system to generate gain or loss of SOX4 in GBM cells. We showed that SOX4 inhibited the growth of GBM cells. A gene expression profiling analysis showed that SOX4 influenced several key pathways including the Wnt/ beta-catenin and TGF-beta signaling pathways. Finally, we showed the activation of p53-p21 signaling and down-regulation of phosphorylated AKT1 by SOX4. These data provide new molecular insights into how SOX4 exerts its functions in glioma cells.

## Methods

### Survival analysis

Z-Scores of mRNA of SOX4 from all three platforms (U133 microarray, Agilent and RNA Seq V2 RSEM) for Glioblastoma Multiforme (TCGA, Provisional) dataset were downloaded using cBioPortal [20,21]. Univariable survival analysis was performed by the Kaplan-Meier method and log-rank test with ‘survival’ R package version 2.37-7 [22].

### Cell lines and cell culture

Human glioma cell lines LN229, T98G, U87MG, U251MG, A172, M059J and M059K were obtained from the American Type Culture Collection. All cells were maintained in Dulbecco’s modified Eagle’s medium (DMEM) with 10% fetal bovine serum (FBS) and 1% penicillin-streptomycin in a humidified incubator with 37°C and 5% CO_2_.

### Plasmid construction, retrovirus infection and transfection

The SOX4 gene from a vector containing SOX4-eYFP (a gift from Carlos S. Moreno, Emory University) was cloned by PCR and inserted into a retrovirus plasmid pBrit-HA/Flag (Plasmid 17519, Addgene, Cambridge MA) between the two *lox P* sites using restriction enzyme BamHI and EcoRI. A 5’ primer containing a Flag tag was used and the final construct was designed to express the Flag-SOX4-HA fusion protein, forward primer (5’-cgcGGATCCgcgATG GATTACAAGGATGACGACGATAAGATGgtgcagcaaaccaaca-3’), reverse primer (5’-ccggaattcGTAGGTGAAAACCAG-3’).

Retrovirus was packaged by co-transfect pBrit-Flag-SOX4-HA/pBrit-Flag-HA together and pcl-Ampho plasmids into 293T cells with the protocol previously described [23], SOX4 overexpression cells (named as LN229_pSFH or A172_pSFH or U87_pSFH respectively for the cell line used) and control cells (named as LN229_con or A172_con or U87_con respectively for the cell line used) were cultured by infecting the respective GBM cell lines with the virus. After 96hours, cells were selected in culture media contains 800ng/ml puromycin (Sigma) for 3 weeks. Survival cells were harvested for functional study and analysis.

For transient transfection, SOX4-HA was cloned to the pCMV-Tag2C vector to generate the Flag-SOX4-HA plasmid. *Cre* recombinase cDNA from the pACN vector [24](a gift from Dr. Mario Capecchi, University of Utah) was cloned into the pEGFP-N1 to construct the *Cre* -GFP plasmid. Flag-HA-GFP plasmid was used as the control. 48 hours after transfection, cells were harvested for further analysis and cell functional test.

### Cell proliferation assay

Cell growth curves were obtained by measuring cell viability with the Cell Counting Kit-8 (CCK-8, Dojindo, Japan) according to the manufacturer’s instruction. 10^3^ cells/well were seeded into 96-well plate on day zero and then cultured for five days. Cell viability was assessed at 6 h (hours), 24 h, 72 h, and 120 h, and normalized to the cell viability measurement at 6 h for each group. Each group was measured in triplicate.

### Cell cycle analysis

For cell cycle analysis, 5 × 10^5^ cells from each sample were trypsinized to make single cell suspension. The cells were then washed with PBS and fixed in 95% ethanol. The cells were treated with RNAse A and stained with propidium iodide (PI) and subjected FACS analysis.

### Colony formation assay

Cells were plated into 6-well plates at 400 cells/well in DMEM culture medium and further incubated for 2 weeks when colonies were sufficiently large for visualization. Colonies were then fixed in methanol and stained with 0.5% crystal violet (Sigma). The stained cells were quantified. Each group was measured in triplicate.

The soft agar assay was performed in 12-well plates containing two layers of Sea Plague Agar (Invitrogen). The bottom layer consisted of 0.8% agar in 1 ml of DMEM with 10% FBS and the top layer consisted 10^3^ cells of stable overexpression cells or control cells. Cells were seeded in each well and cultured for three weeks. Colonies were photographed under a microscope and counted.

### Reverse transcription and real-time PCR

RNAs from cell lines were extracted using TRIZOL (Invitrogen) and 1μg of total RNAs was reverse transcribed into cDNAs using ReverTra Ace qPCR RT Kit (Toyobo, Japan). For quantitative real-time PCR, 20ng of cDNAs was used as template using the SYBR® Premix Ex Taq™ II (Taraka, Japan). GAPDH was used as control. Primers for target genes were listed in (Additional file 1: Table S1). Relative expression was calculated as described [25].

### Immunoblotting and analysis

Cells were washed twice with PBS and lysed in RIPA buffer (25mM Tris HCl, pH 7.6, 150mM NaCl, 1% NP-40, 1% sodium deoxycholate, 0.1% SDS, 1mM PMSF [P7626, Sigma] with the Protease Inhibitor Cocktail [Roche]). Nuclear and cytosolic fractions were extracted using a nuclear protein extraction kit (Beyotime, Jiangsu, China) according to the manufacturer's instructions. Protein concentration was determined using the BCA protein kit (23225, Thermo, USA). Total cell lysate (30 μg) and nuclear or cytosolic fractions (15 μg) were loaded on SDS-PAGE and electrophoretically transferred to polyvinylidene difluoride membrane (Milipore, Bedford, MA). The membranes were blocked with 5% milk protein in TBST (0.6% Tween, 10 mM Tris pH7.6 and 150 mM NaCl in H_2_O) and incubated with primary antibodies at 4°C overnight followed by incubation with secondary antibody for 1h at room temperature before exposure. Primary antibodies used were: anti-HA antibody (1:10000, ab9110, Abcam), anti-beta-catenin antibody(1:2000, ab32572, Abcam), anti-p53 antibody (1:1000, ab32389, Abcam), anti-AKT1 antibody(1:500, ab81283, Abcam), anti-AKT1p antibody(1:500, ab81283, Abcam), anti-p21 antibody(1:500, AM2385, Abzoom),anti-Ki67 antibody(1:250, ab92742, Abcam), anti-GAPDH antibody (1:2500, ab9485, Abcam), anti-Histone H3 antibody (1:2500, 2134–1,Epitomics), Anti-beta Catenin (phospho S33 + S37) antibody (1:1000, ab75777, Abcam).

### Expression profiling and Gene Ontology analysis

In order to identify genes subjected to SOX4’s regulation in GBM cells, total RNAs from SOX4 overexpression stable cell lines LN229_pSFH and LN229_con were used for expression profiling using the PrimeView Human Gene Expression Array (Affymetrix) using a standard Affymetrix protocol. The array data were submitted to the GEO database (GSE51301). Gene Ontology analysis was done using the DAVID program (http://david.abcc.ncifcrf.gov/) [26,27].

### Statistical analysis

Statistical analysis was performed using the Student’s t-test. P <0.05 was considered to be statistically significant. The results are reported as mean ± SD.

## Results

### High SOX4 expression was significantly associated with good prognosis of primary GBMs

**Figure 1:**
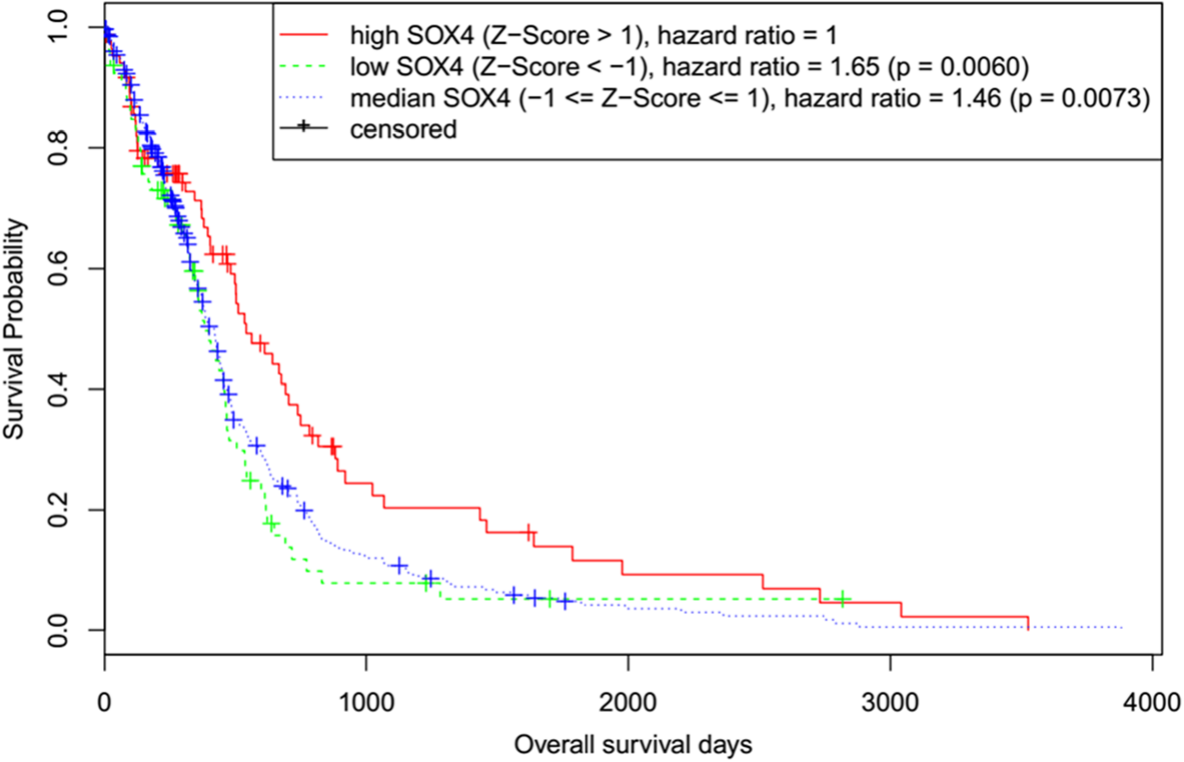
**Kaplan-Meier analysis comparing SOX4 mRNA status with survival in primary GBM samples.** Z-Scores of mRNA of SOX4 (TCGA, Provisional) were downloaded using cBioPortal. Kaplan-Meier survival analysis of SOX4 expression in primary GBM showed the overall survival status was significantly different between the groups with different SOX4 expression level. The hazard ratio of low-SOX4(Z-Score < 1) or median-SOX4 (Z-Score = 1)patients was larger than high-SOX4(Z-Score > 1)with p-value < −0.01.

We have previously reported that SOX4 is over-expressed in primary GBM tissues compared to normal brain tissues [8]. In this study, we analyzed the expression level of SOX4 and its association with GBM patient survival using the data from TCGA (Additional file 2: Table S2) including data from all three analytical platforms (U133 microarray, Agilent and RNA Seq V2 RSEM). We downloaded the data using cBioPortal [20,21]. The expression levels of the SOX4 in the downloaded data were computed and represented as the Z-scores, which are the number of standard deviations away from the mean of expression in the population. 85 patients with SOX4 mRNA Z-Scores >1 were grouped into the high-SOX4 group, and 79 patients with Z-Scores < −1 into the low-SOX4 group. The remaining 331 patients were regarded as the median-SOX4 group. Using Kaplan-Meier survival analysis, we found that the overall survival status was significantly different between the groups with different SOX4 expression level (logrank test p-value =0.0104). The hazard ratio of the low-SOX4 is 1.65, and the hazard ratio of the median-SOX4 group is 1.46, which are both significantly greater compared with the hazard ration of 1 in the high-SOX4 group (P values of 0.006 and 0.0073 respectively (Figure 1), indicating that high SOX4 expression is strongly associated with the good prognosis of GBM patients.

### Overexpression of SOX4 reduces cell proliferation and colony formation in GBM cells

To understand the protective mechanism of goof prognosis of high expression SOX4 for GBM patients, we decided to overexpress the SOX4 gene in GBM cell lines and to assess its impact on cell proliferation and colony formation. In order to find a suitable GBM cell lines for overexpression SOX4, we compared the SOX4 expression at mRNA levels for seven GBM cell lines LN229, T98G, U87, U251, A172, M059J and M059K by real-time PCR (Additional file 3: Figure S1). We found that GBM cell line LN229 have relative lower expression of SOX4 than others.

**Figure 2:**
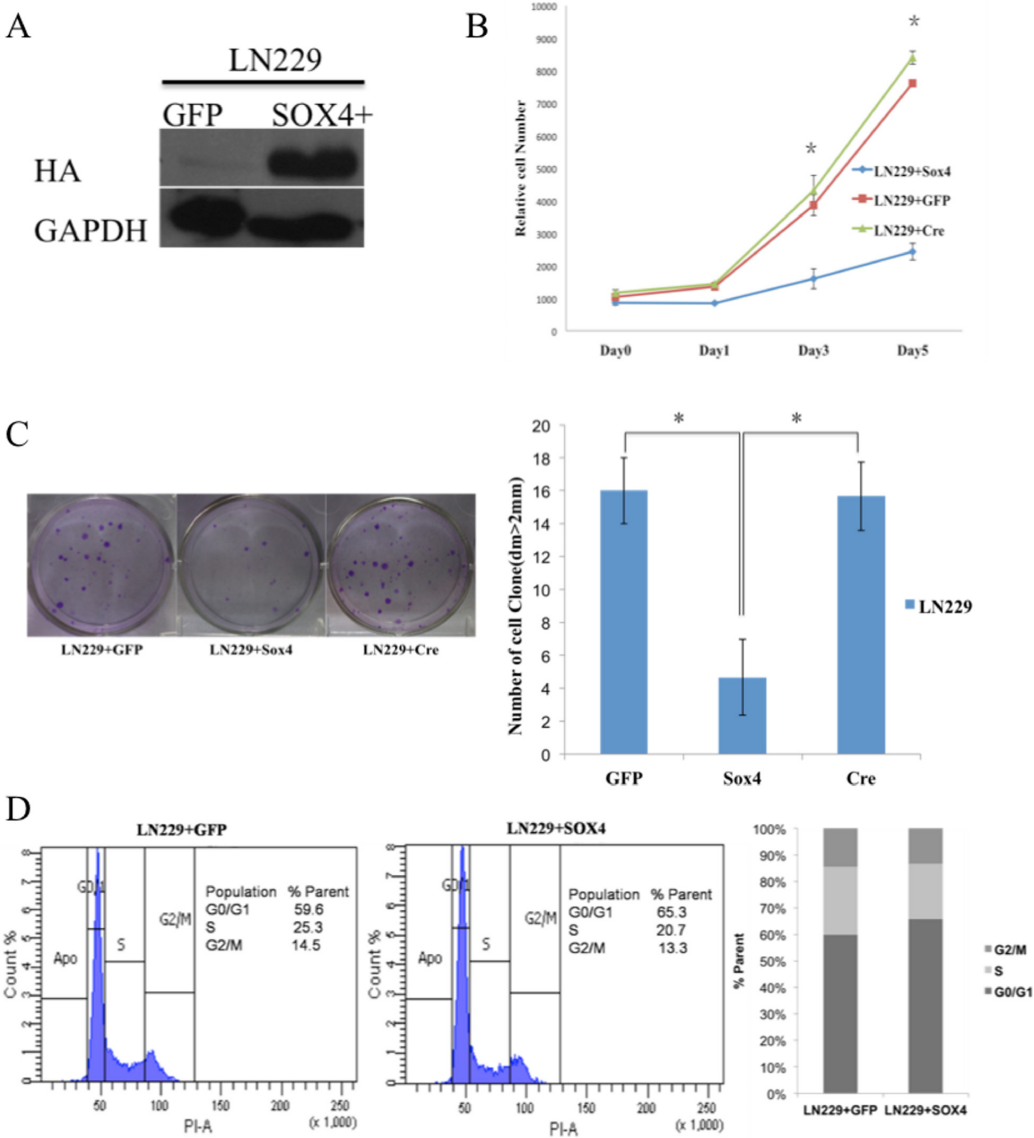
**Transient SOX4 overexpression inhibits growth of LN229 cells and induces G0/G1 cell cycle arrest. (A)** LN229 cells were transiently transfected with Flag-Sox4-HA or pEGFP-N1 plasmid as control. After 48 hours, protein expression was assayed by Western blotting using anti-HA antibody **(B)** Cell vitality was determined by CCK-8 assay after transfection of LN229 cells with Flag-Sox4-HA plasmid or pEGFP-N1 and *Cre*-GFP as control at the indicated time points. Values at the indicated time points were provided as the mean cell number with an SD of eight wells. *, P < 0.05 **(C)** Colonies formed by Flag-Sox4-HA or pEGFP-N1or *Cre*-GFP infected LN229 cells were shown 2 weeks after plating in 6 well plate. Upper panel showed the quantification of the colony in Flag-Sox4-HA or pEGFP-N1or *Cre*-GFP infected LN229 cells. Values are the means ± SD of triplicate experiments, *P < 0.05 **(D)** Impact of SOX4 on cell cycle of LN229 cells. The percentage of cells in G0/G1, S, and G2/M phases is shown in the left panel. And the statistic analysis is also shown in the right panel.

We started with testing the functional consequences of SOX4 overexpression using a transient expression system. We transiently transfected GBM cell line LN229 with the Flag-SOX4-HA construct and we used the empty vector pEGFP-N1 construct without the HA tag as the control. Forty-eight hours after transfection, we detected marked increase of SOX4 protein expression in LN229 cells by western blot with anti-HA antibody (Figure 2A), as shown by the band at 70 KDa for the Flag-SOX4-HA protein [10,28].

By cell proliferation assay, we found that LN229 cells with transient SOX4 overexpression grew significantly slower than cells with the control construct (Figure 2B). In addition, SOX4 overexpression cells lost the ability to form colonies (Figure 2C). FACs analysis showed an increased accumulation of SOX4 overexpression LN229 cells in the G0/G1 phase (65.3%) compared with the cells with Flag-HA-GFP control (59.6%), p < 0.05 (Figure 2D).

**Figure 3:**
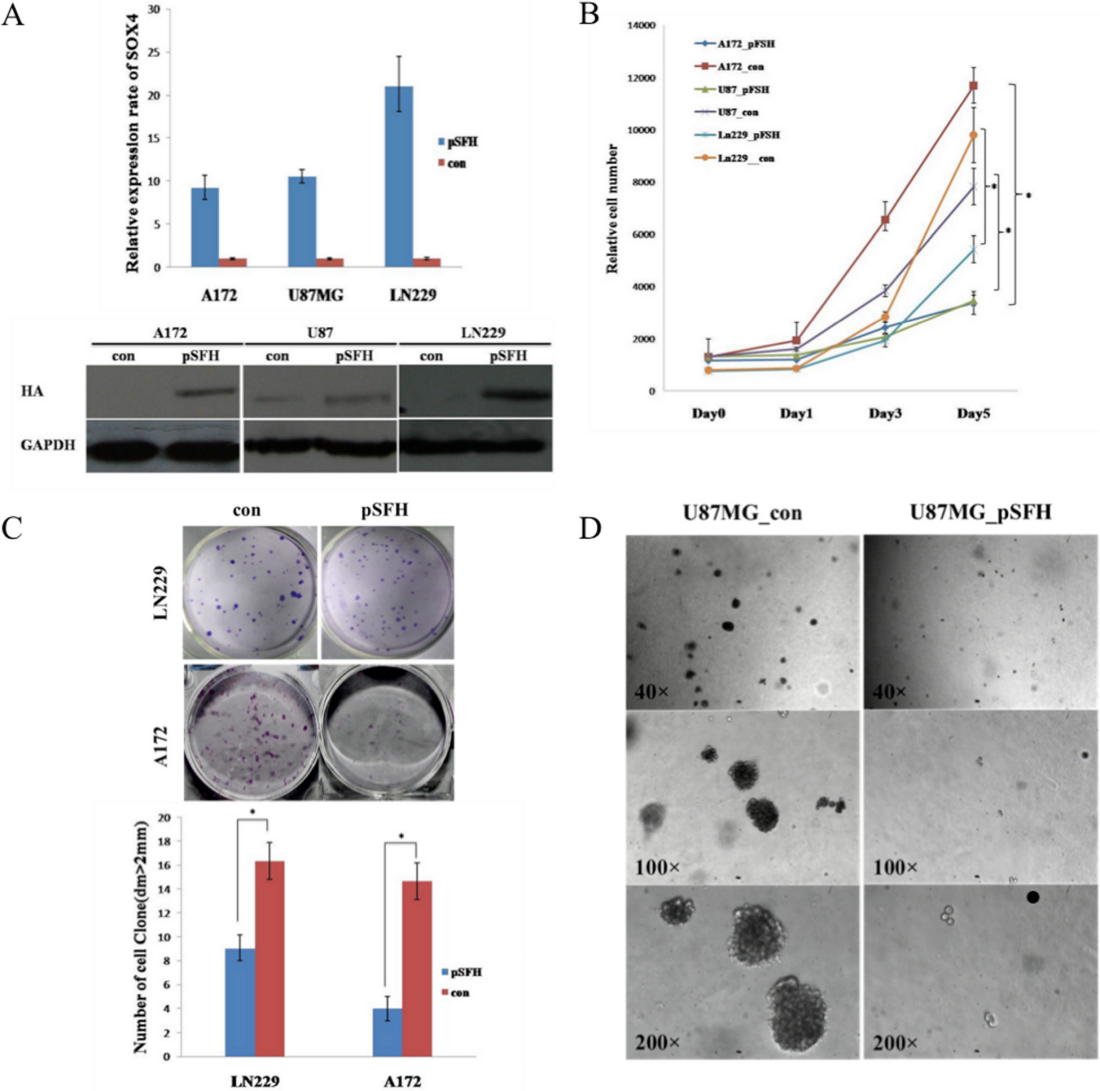
**Overexpression of SOX4 reduces proliferation and colony formation of GBM cells. (A)** Validation of SOX4 expression in stable SOX4 overexpression cells (LN229_pSFH/A172_pSFH/U87_pSFH) and control cells (LN229_con/A172_con/U87_con) by Real-time PCR and Western blot with anti-HA antibody. **(B)** The vitality of GBM cells stably express Flag-SOX4-HA or GFP was determined by CCK8 assay. Values at the indicated time points were provided as the mean cell number with an SD of eight wells. *, P < 0.05 **(C)** Colonies formed by LN229_pSFH/A172_pSFH or LN229_con/A172_con were shown 2 weeks after plating in 6 well plate. Right panel showed the quantification of the colony formation. Values are the means ± SD of triplicate experiments. *, P < 0.05 **(D)** Colonies formed by U87_pSFH or U87_con were shown 3 weeks after plating in Soft agar.

Encouraged by our data from the transient analysis, we decided to stably over express SOX4 in GBM cells. In order to easily remove the SOX4 in the overexpression construct as a way to study functional reversal, we decided to use a *Cre*/lox P recombination system to engineer SOX4 overexpression. GBM cell lines LN229, A172, U87MG were selected to over-express Flag-SOX4-HA by retroviral infection. After selected with 800ng/ml puromycin for three weeks, cells were harvested and their expression of exogenous Flag-SOX4-HA were validated by real-time PCR and western blot (Figure 3A).

SOX4 overexpression cells in all of the three cell lines LN229, A172, U87MG showed reduced cell proliferation with stable SOX4 expression (Figure 3B). LN229_pSFH and A172_pSFH showed reduced colony formation capabilities compared with LN229_con and A172_con cells (Figure 3C). For U87 GBM cell lines, because SOX4 overexpression changed its morphology (Additional file 4: Figure S2), we did soft agar assay instead. After being cultured in suspended condition for three weeks, colonies of U87_con were visible by eyes while U87_pSFH could not form sphere from single cell (Figure 3D). In summary, SOX4 overexpression inhibits cell proliferation in GBM cells and also blocked the anchorage-independent growth ability of U87 GBM cells.

### Knock out SOX4 by *Cre* recombinase promotes cell proliferation in LN229 cells

**Figure 4:**
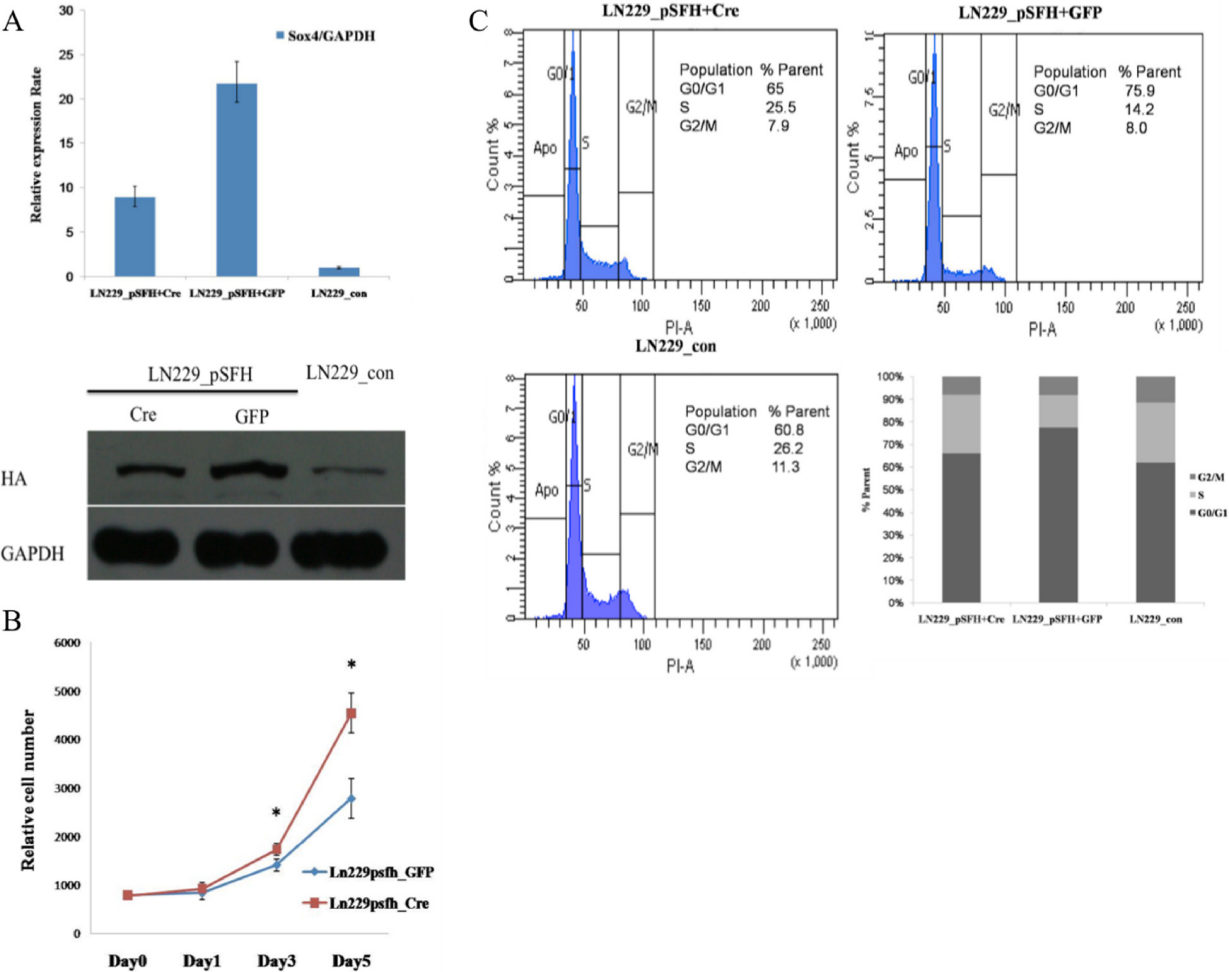
**Knock out SOX4 by** ***Cre*** **recombinase restored cell growth and colony formation. (A)** Validation of SOX4 expression in Flag-SOX4-HA stable expressed cells transfected with *Cre*-GFP or pEGFP-N1 by Real-time PCR and Western blot with anti-HA antibody **(B)** Cell vitality was determined by CCK-8 assay after transfection of Flag-SOX4-HA stable expressed cells with *Cre*-GFP or pEGFP-N1 as control. Values at the indicated time points were provided as the mean cell number with an SD of eight wells. *P < 0.05 **(C)** The reduction of SOX4 reduced cells accumulated in G0/G1 phase. The percentage of cells in G0/G1, S, and G2/M phases is shown in the left panel. And the statistic analysis is also shown in the right panel.

To see whether the effect of SOX4 overexpression on cell proliferation is reversible, we took advantage of the loxP system in our construct where the SOX4 gene can be removed after introducing *Cre* recombinase to the cells. To estimate the potential influence of *Cre* recombinase on cell, we did transient transfection of *Cre*-GFP construct into LN229 cells and no significant changes on cell proliferation or colony formation ability of LN229 cells were found (Figure 2B and C). 48 hours after transfection of *Cre*-GFP plasmid, exogenous SOX4 expression was reduced about 50% in LN229_pSFH cells with *Cre* recombinase compared with the control cells without *Cre* (transfected with the pEGFP-N1 plasmid instead) (Figure 4A), and the cell proliferation increased dramatically at day 5 in LN229_pSFH cells with *Cre* recombinase compared to that for the control cells (Figure 4B). Flow cytometry analysis showed a decreased accumulation of cells in the G0/G1 phase (65%) for pSFH cells transfected with *Cre*-GFP compared with that for pSFH cells transfected with pEGFP-N1 (75.9%), although pSFH cells transfected with *Cre* still have higher number of cells at G0/G1 phase (65%) compared with that in LN229_con cells (60.8%) (Figure 4C). These data suggested that SOX4 suppressed GBM cell proliferation by inducing G0/G1 cell cycle arrest.

### Global expression profiling of SOX4 regulated genes

**Figure 5:**
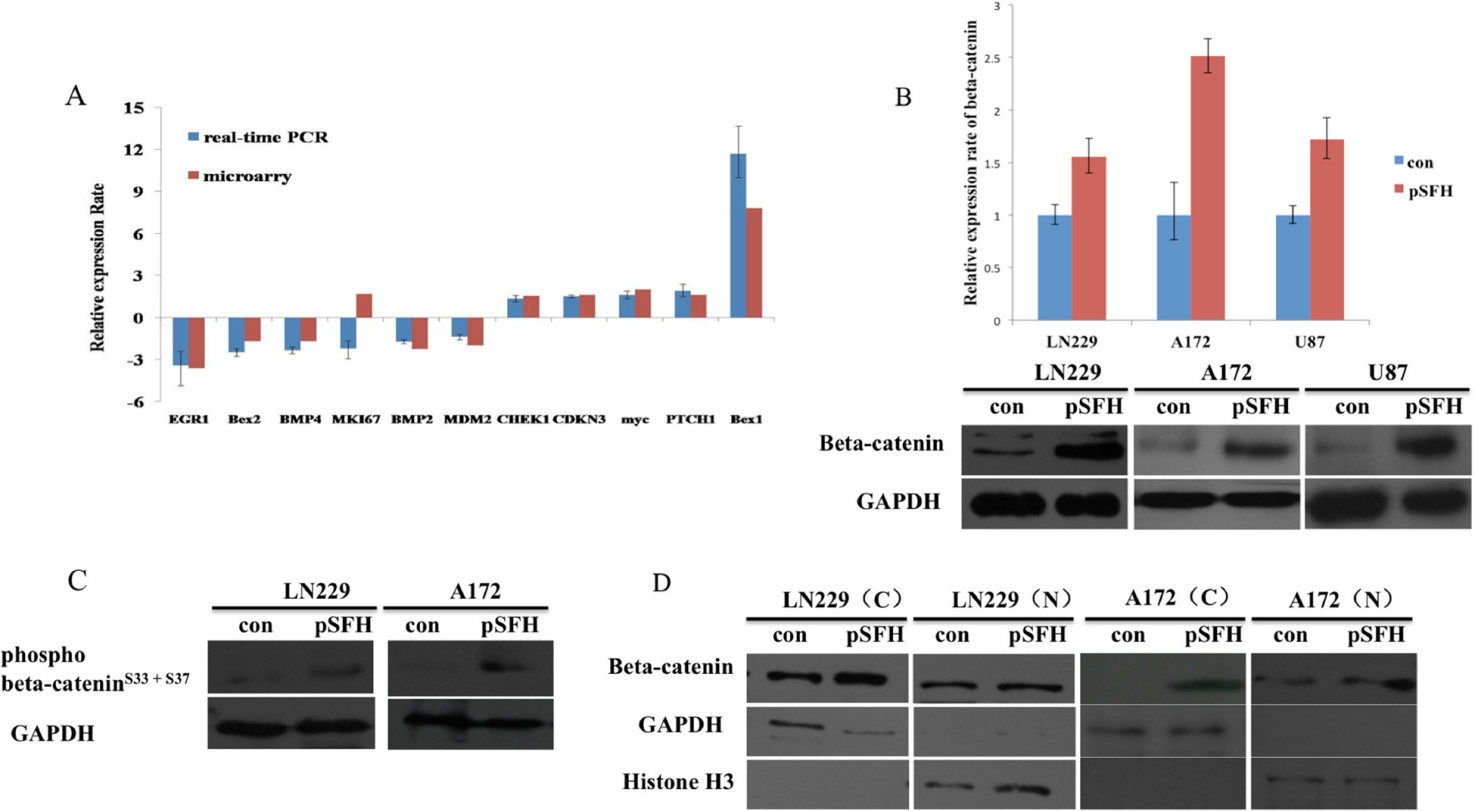
**Microarry analysis of genes regulated by SOX4 in LN229 SOX4 overexpressed cells. (A)** Validation of SOX4 regulated gene by Real-time PCR. The relative expression of target gene was normalized to the endogenous control GAPDH. Three replicate PCR were performed and the standard errors of the mean were indicated by error bars. **(B)** Validation of SOX4 increased beta-catenin in LN229_pSFH/A172_pSFH/U87_pSFH compare to LN229_con/A172_con/U87_con cells by RT-PCR and Western blotting with anti beta-catenin antibody. **(C)** Validation of SOX4 increased beta-catenin in LN229_pSFH/A172_pSFH compare to LN229_con/A172_con by Western blotting with anti phosphor-beta-catenin antibody. **(D)** Validation of cellular localization of SOX4 increased beta-catenin. Anti-histone H3 antibody was used to normalize the amount of nuclear sample.

To identify genes regulated by SOX4 in GBM cells, we used Affymetrix’s The GeneChip® PrimeView™ Human Gene Expression Array to compare SOX4 overexpression LN229_pSFH and LN229_con cells. We identified 633 genes were changed more than 1.8 fold (Additional file 5: Table S3). The array data was confirmed by doing real-time PCR, and 9 of 10 randomly selected changed genes identified by array was confirmed by RT-PCR (Figure 5A).

We performed GO analysis with 633 SOX4 regulated genes and found that they are enriched in biological progression contains GO:0007049_cell cycle, GO:0006260_DNA replication, GO:0006974_response to DNA damage stimulus, GO:0007017 ~ microtubule-based process, GO:0007010 ~ cytoskeleton organization, GO:0000075 ~ cell cycle checkpoint with p-value < 0.001, FDR < 0.05 (Table 1). We also compared our list of SOX4 regulated genes in GBM cells to a previously published list of SOX4 regulated genes in prostate cancer cells [23], and we found that 54 genes are commonly changed in both glioma and prostate cancer in response to SOX4, with 33 genes up-regulated and 14 genes down-regulated genes (Additional file 5: Table S3).

**Table 1:** **Biology process enriched in SOX4 regulated gene in LN229_pSFH cells by GO analysis**

**GO**	**Annotation**	**Count**	**P Value**
GO:0007049	Cell cycle	187	8.05E-40
GO:0006260	DNA replication	59	4.30E-18
GO:0006974	Response to DNA damage stimulus	78	5.92E-13
GO:0033554	Cellular response to stress	96	3.11E-10
GO:0007010	Cytoskeleton organization	73	9.30E-08
GO:0008283	Cell proliferation	68	3.62E-06
GO:0010941	Regulation of cell death	100	4.56E-04
GO:0006916	Anti-apoptosis	33	0.001012965
GO:0045596	Negative regulation of cell differentiation	34	0.001139053

SOX4 was previously shown to enhance beta-catenin/TCF activity and the proliferation of colorectal cancer cells [29,30]. Overexpression of SOX4 in endometrial carcinomas cell lines caused enhancement of beta-catenin/TCF4-driven transcription, whereas cells stably overexpressing SOX4 demonstrated a low proliferation rate, through transactivation of the p21 gene [31]. From our microarray data, we also found that beta-catenin is up-regulated by SOX4 (Additional file 5: Table S3) in GBM cell line LN229. We further confirmed that beta-catenin is up-regulated by SOX4 by RT-PCR and western blot in all of the three GBM cell line LN229, A172 and U87 (Figure 5B). Additionally, Ser 33 and Ser 37 phosphorylation of beta-catenin by GSK3B was also increased (Figure 5C). Western blot analysis with nuclear and cytoplasmic fractions of cellular proteins indicated a strong increasment of beta-catenin in the cytoplasm but little if any changes in the nuclear after SOX4 overexpression (Figure 5D).

### SOX4 reduces cell proliferation and causes cell cycle arrest via Akt-p53 axis

**Figure 6:**
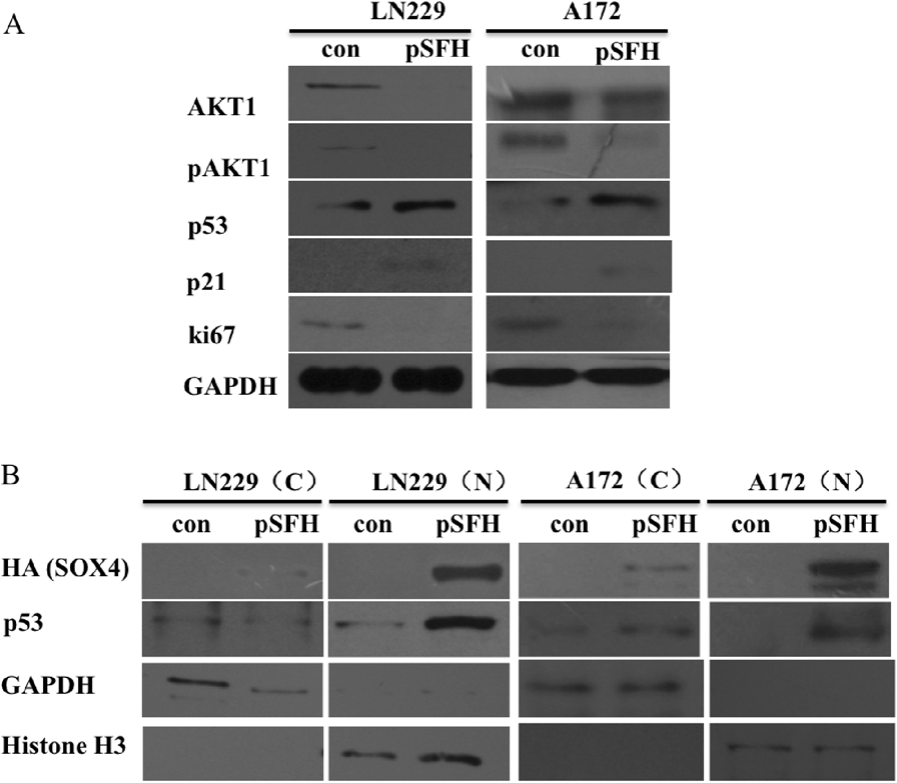
**Validation of genes regulated by SOX4. (A)** Validation expression of p53, AKT1, AKT1p, p21 and Ki67 in Flag-SOX4-HA stable expressed cells compare to control cells using western blot **(B)** Validation of cellular localization of SOX4 and p53. Anti-histone H3 antibody was used to normalize the amount of nuclear sample.

Previously studies have established that SOX4 interacts with and stabilizes p53 protein, a key tumor suppressor for cancers [14,32-34]. To assess whether p53 is involved in SOX4 mediated inhibition of cell proliferation and cell cycle arrest in GBM cells, we determined the expression of p53 and its downstream target p21, which is associated with growth arrest [35], by Western blot. We found that both p21 and p53 proteins were increased by SOX4 (Figure 6A). We further showed that both SOX4 and p53 proteins were accumulated in the nucleus after SOX4 overexpression in GBM cells (Figure 6B).

Akt enhances Mdm2-mediated ubiquitination and degradation of p53 [36]. We therefore analyzed Akt and phosphorylated Akt expression after SOX4 overexpression and found that both the protein levels of AKT1 (official gene name for Akt) and phosphorylated AKT1p were down-regulated by SOX4 overexpression (Figure 6A). We showed earlier with the microarry data that MDM2, a key modulator of p53 protein levels in cells by binding to p53 and promotes p53 degradation via the ubiquitin/proteasome pathway, is downregulated by SOX4 in LN229 (Figure 5A). Furthermore, Ki67, a proliferation marker [37], was also found to be reduced in SOX4 overexpression GBM cells at both the mRNA (Figure 5A) and the protein levels (Figure 6A). Thus it seemed that SOX4 induced p53 in the nucleus is partially caused by down regulation of AKT1 and reduced phosphorylated AKT1p.

## Discussion

We reported our finding that high SOX4 expression was significantly associated with good prognosis of primary GBM patients (Figure 1). However, overexpression of SOX4 in GBM cell lines inhibited cell proliferation and G0/G1 cell cycle arrest (Figures 2 and 3). These seemly contradictory observations between observation in primary clinical samples and in vitro cell line functional analysis is similar to what Aaboe et al. observed in bladder cancer [4]. They found that SOX4 is over expressed in bladder cancer tissues compared to normal tissues, but strong SOX4 expression was found to be correlated with increased patient survival (P <0.05) of bladder cancer patients [4], and when introduced to bladder cancer cell line HU609, it reduced cell viability by promoting apoptosis and necrosis [4]. The exact mechanism of this seemly contradict phenomena need further investigation. Sox factors function as either tumor activators or repressors depending on the cellular context and associated interacting proteins [38]. One of the differences between the cell lines we used and the GMB tumor tissues is the P53 mutation status. P53 are often mutated in GBM tumors, seen in 25–30% of primary GBM and 60–70% of secondary GBM [39]. However, the GBM cell line LN229, A172 and U87 that we used in the study turned out to contain wide type p53 [40]. One possible mechanism is that SOX4 acts differently on the wild-type P53 than the mutated P53. Further experimentation is needed to verify this speculation.

The p53 protein is an important tumor suppressor that is inactivated in most tumors. It controls growth arrest and apoptosis by its transcriptional activation and repression of target genes [41]. p53 is thought to exert its function in G1 checkpoint control mainly through increased expression of the p21 gene [42]. We found p53 were increased and were accumulated in the nucleus with SOX4 (Figure 6B), while p53’s downstream target p21 was up-regulated (Figure 6A), and leading to G0/G1 cell cycle arrest (Figure 4C). In addition, SOX4 reduced expression levels of AKT1 and phosphorylated AKT1 (Figure 5A), which could enhance Mdm2-mediated ubiquitination and degradation of p53 [36,43]. Thus, we propose a model to explain SOX4’s action in GBM cell lines: SOX4 could down-regulate Akt, which might result in increased stability of p53, as shown by increased p53 protein expression in SOX4 overexpressed GBM cell lines and induced p21 signal which leading to G0/G1 cell cycle arrest.

SOX4 has been proposed either as an oncogenor or a tumor suppressor. Our finding that SOX4 acts as a tumor suppressor in GBM is consistent with previously reported roles of SOX4 as a tumor suppressor. For example, SOX4 induces cell apoptosis through caspase activation in hepatocarcinoma cells and HeLa cells [14,15]. Nuclear overexpression of SOX4 in HCC samples is correlated with diminished risk of recurrence and improved overall survival time in HCC patients [32]. Pan *et al.* reported that SOX4 promotes cell cycle arrest and apoptosis, and inhibits tumorigenesis by blocking Mdm2-mediated p53 degradation and facilitating p300/CBP/p53complex formation [34]. In lung cancer cell line H1299, SOX4 increases PUMA expression in response to trichostatin A (TSA) and induces apoptosis in a p53 independent manner [44].

Our data proved that SOX4 inhibited cell growth and induced G0/G1 cell cycle arrest in GBM cells. We found that AKT1 and AKT1p were reduced while SOX4 overexpression in GBM and p53 protein level was increased and co-localized in nuclear with SOX4. However, SOX4 increased beta-catenin expression seems to limited to the cytoplasm and failed to initiate cytoplasm-to-nuclear translocation of beta-catenin, suggesting the action of SOX4 seems unlikely to involve directly transcriptional changes of beta-catenin directly regulated genes.

Microarray analysis showed that SOX4 regulated genes were enriched in Gene Ontology terms related to cell cycle progression (Table 1 and Additional file 5: Table S3), consistent with our functional assay showing SOX4’s role in cell cycles. We also identified many interesting genes regulated by SOX4, suggesting SOX4’s diverse roles in cancer cells. For example, we found SOX2 is upregulated by SOX4 (Additional file 5: Table S3), which is consistent with previous findings that the transforming growth factor-β (TGF-β)-Sox4-Sox2 pathway is essential for glioma-initiating cells to retain their stemness [18,19]. We also identified several other genes involved in TGF-beta signaling include BMP2 and SOX10, whose expression were also changed after SOX4 overexpression (Additional file 5: Table S3).

Finally, we demonstrated that beta-catenin was upregulated by SOX4 (Figure 5B-D), suggesting a role of SOX4 in WNT signaling in GBM cells. SOX4 was previously shown to enhance beta-catenin/TCF activities in several cancer types [29-31]. p53 can reduce the proliferation-supporting effect of beta-catenin/TCF signaling by downregulating Tcf-4 expression even with transcriptional activation by beta-catenin in the nucleus [45]. Considering SOX4 increased both p53 and beta-catenin in GBM cells, there might be a cross talk between these two signals.

## Conclusions

We showed that high SOX4 expression was significantly associated with good prognosis of primary GBM patients, suggesting the SOX4 might be a prognosis marker for GBM. However, in cell line models of GBM, SOX4 seems to behave as a tumor suppressor, a discrepancy to the good prognosis offered by the high SOX4 expression. Further investigation is necessary to understand this discrepancy and to determine the different cellular contexts in cancer cells that turn SOX4 into a tumor suppressor or tumor promoter.

## Additional files

Additional file 1: Table S1. Primers used for Real-time PCR. Additional file 2: Table S2. Clinical data of primary GBM tissues downloaded from TCGA. Additional file 3: Figure S1. The quantitative RT-PCR results of SOX4 mRNA expression in GBM cell lines. Additional file 4: Figure S2. Morphological change of U87 after SOX4 over expression in colony formation assay. Additional file 5: Table S3. 633 genes regulated by SOX4 in LN229 cells and its overlap between genes regulated in LNCaP prostate cancer cells.
