# Supplementary figures and images for: SOX4 inhibits GBM cell growth and induces G0/G1 cell cycle arrest through Akt-p53 axis
Source: BMC Neurol. 2014 Nov 1;14:207. doi: 10.1186/s12883-014-0207-y (PMC4233052; doi:10.1186/s12883-014-0207-y)

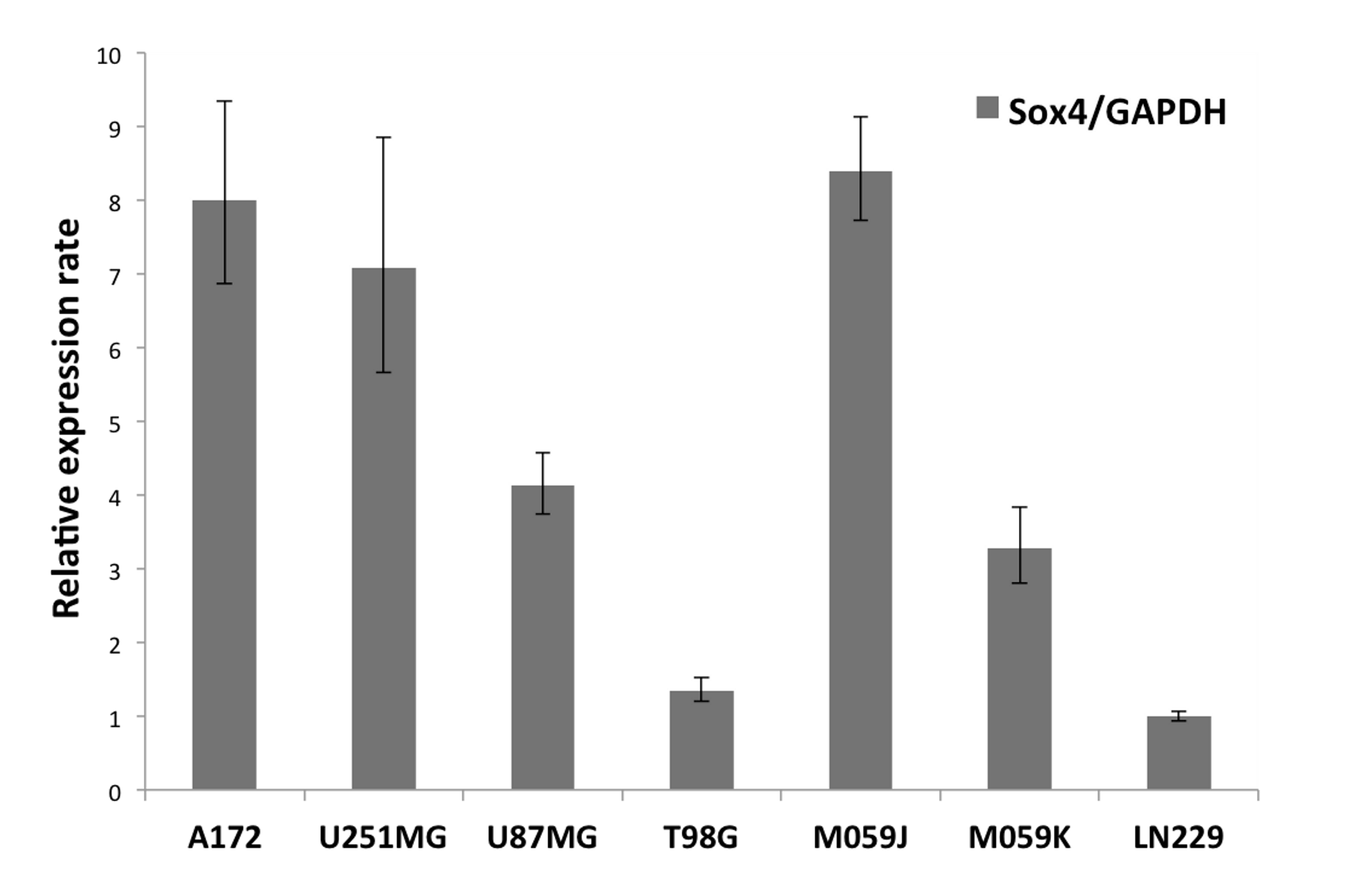

Supplement: Additional file 3: Figure S1. — The quantitative RT-PCR results of SOX4 mRNA expression in GBM cell lines. [file 12883_2014_207_MOESM3_ESM.tiff]

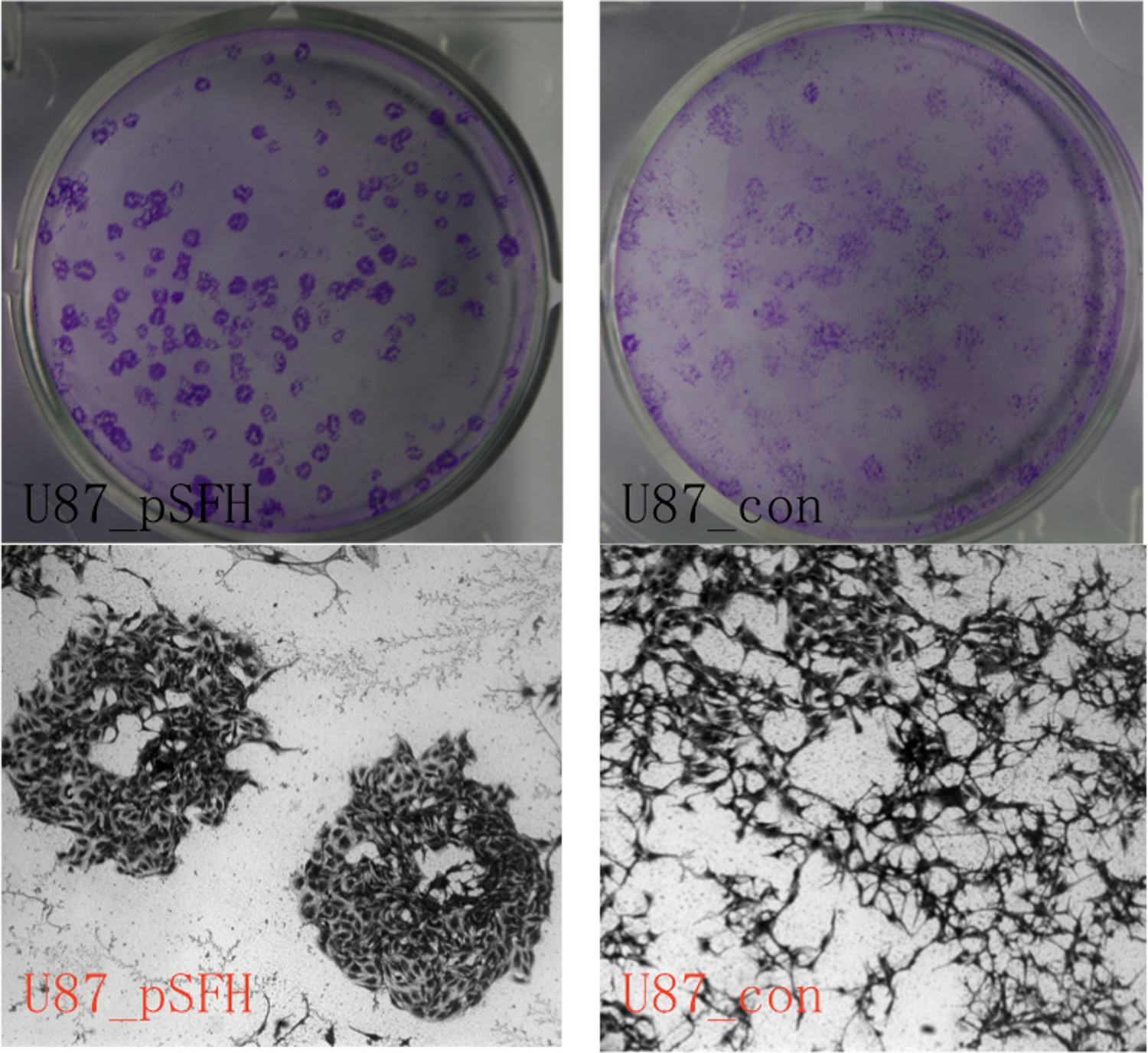

Supplement: Additional file 4: Figure S2. — Morphological change of U87 after SOX4 over expression in colony formation assay. [file 12883_2014_207_MOESM4_ESM.tiff]
